# The future of public health: the importance of workforce

**DOI:** 10.1186/1743-8462-6-4

**Published:** 2009-04-09

**Authors:** Vivian Lin, Rebecca Watson, Brian Oldenburg

**Affiliations:** 1 School of Public Health, La Trobe University, Bundoora, Australia; 2 Australian Insitute of Health Policy Studies (AIHPS), Monash University, Melbourne, Australia; 3 School of Public Health and Preventive Medicine, Monash University, Melbourne, Australia

## Abstract

Health workforce has become a major concern and a significant health policy issue around the world in recent years. With recent international and national initiatives and models being developed and implemented in Australia and other countries, it is timely to understand the need and the rationale for a better trained and educated public health workforce for the future. Much more attention should also be given to evaluation and research in this field.

Through this thematic series on Workforce and Public Health, we have drawn on the diverse nature of public health, workforce implications, education and training and national and international case examples of ongoing improvements and issues in this sector.

## Why a special call for articles on public health workforce?

Health workforce has become a major concern and a health policy issue around the world in recent years and it was the focus for the World Health Report in 2006 [1]. Globally, increased concerns about the 'brain drain' of health professionals from developing countries to more developed countries led to the draft WHO code of practice about human resources for health [2]. Within Australia, the release of the Productivity Commission Report in 2005 [3] focused national attention on the significance and importance of the current and future health workforce in this country. This culminated in the signing of an inter- governmental agreement to move to a national registration system in Australia for a number of the health professions in March 2008. This was followed by a more recent appointment of a management committee for the Australian Health Practitioner Regulation Agency which will be established. Other important and related issues being progressed nationally include the development of competencies for a number of health professions and the piloting of new workforce models [4].

However, all of these initiatives have been silent on issues to do with what kind of workforce is required to improve Australia's public health workforce and thereby, address future public health issues and challenges, including those challenges that are not currently known. With the advent of a new Labor government in late 2007, which has seen the establishment of a number of new commissions, committees and other working groups including – the National Health and Hospital Reform Commission, Preventative Health Taskforce, Primary Health Care Strategy, National Indigenous Health Equity Council, there has been considerable discussion regarding an increased emphasis on disease prevention and health promotion. However, for such policy intent to be translated successfully into practice, this will rely on having a workforce that has relevant and specialised knowledge and skills related to the field of prevention and public health practice more generally. Therefore, it is timely to examine what kind of workforce will be required and to consider the related policy agendas that require attention.

## What is the public health workforce?

If public health is defined as "the organized efforts of society to keep people healthy and prevent injury, illness and premature death [5]", then the public health workforce includes those professionals and other workers who are engaged in these efforts. Such a definition immediately leads to a range of challenging questions – How is it enumerated? What are the required qualifications? What is the skill set? What does it actually do? These problems of workforce definitions, and related issues of workforce planning, have challenged all countries and were given great salience during the SARS pandemic in 2003 [6].

Historically, the public health workforce has been seen as 1) those who are involved with public health and related programs, typically in the public sector, and/or 2) those that have some kind of specific training or degree in public health, such as a Master of Public Health (MPH) or a qualification in a related discipline. However, it is also the case that the majority of those who work in such areas do not possess a formal qualification in public health [7]. While Australia's National Public Health Partnership [8] defined several categories of public health workforce a number of years ago – including those who are specialists, those in the health sector who incorporate public health practice into their normal clinical work or other roles, and those in other sectors whose work contribute to the health of the public it – is the MPH which has had the historical and global recognition as the basic qualification for the public health professional, with the DrPH and PhD as research degrees. In more recent times, there has been an increase in the number of specialist qualifications as the number of career pathways into and beyond public health have expanded. This has spawned the development of a range of public health sub-specialties including epidemiology, biostatistics, health promotion, environmental health, health economics, health policy, and health services management. The growth in postgraduate specialisation in public health has also been accompanied by the commensurate development of undergraduate degrees in public health and health promotion as well.

## Policies affecting public health workforce development in Australia

The growth of public health education in Australia has been a product of purposeful government investment as well as the result of broader policies in the health and education sectors. However, there are no currently coordinated efforts underway to address these issues for the future [9].

The proliferation of public health education in Australia has been a relatively recent phenomenon, after decades of institutional stability (or stasis). The original School of Public Health and Tropical Medicine was established by the Commonwealth Institute of Health at the University of Sydney in 1927. For much of the 20^th ^century, Australians seeking an MPH qualification had to relocate to Sydney for 2 years to undertake this postgraduate training. The 1988 Kerr White Report (the Bicentennial Review of Public Health Education and Research) led to the establishment of Public Health Education and Research Program (PHERP) and the MPH became available in most faculties of medicine around Australia by the early 1990s. The subsequent Dawkins reforms in higher education (of a 'national unified system') also led to the flourishing of public health education in many new faculties of health sciences in universities that did not have medical schools. By the end of the 20^th ^century and for the first time in Australia, MPH and other undergraduate and postgraduate programs in public health and related fields, such as health promotion, were established and built an identity quite separate from the field of medicine.

In late 1993, the Commonwealth exercised its policy purchasing power by stipulating that PHERP funding would be available to consortia of universities in each state, so students could access the full range of public health disciplines. Further reforms in higher education, along with the development of the National Competition Policy, however, continued to reshape the landscape for universities. In the decade since the late 1990s, universities began to shorten the length of MPH programs, offer more specialised postgraduate qualifications, introduce undergraduate qualifications in public health, as well as professional doctorates (by research and by coursework) in public health. International students grew in numbers for the MPH as universities actively recruited for full-fee paying students. These trends were reinforced, if not exacerbated, by the Commonwealth attempting to purchase new "products" (i.e. so-called "PHERP Innovations") through "top-slicing" of existing dollars for new projects while the funding base was locked into historical patterns.

Concurrently, the states of NSW and Victoria began to offer advanced practitioner training programs in public health for those who had completed MPHs. These placement-based schemes generated a group of well-trained public health professionals who have largely fast-tracked into management and leadership roles. At the other end of the workforce spectrum, with the growth of community-based health services, population health competencies were developed for the vocational education and training (VET) sector as well [10]. These training programs became available for such workforce categories as drug and alcohol workers, Aboriginal health workers, home care attendants. Finally, as a consequence of the PHERP Review in 2005 [11] which placed the issue of the quality of MPH education on the policy agenda, draft practice-based competencies are currently being finalised as part of the proposed revision for MPH programs.

By 2008, there were at least 21 higher education institutions in Australia which were offering undergraduate and/or postgraduate qualifications in public health, with 17 institutions receiving at least some funding through the Commonwealth PHERP scheme.

## Global expansion in workforce development in public health

These Australian developments in public health workforce education are occurring as other countries are also reviewing their policies and frameworks. The US Institute of Medicine released a major report on the future of the public health workforce in 2002 [12] and the US Association of Schools of Public Health jointly with their Council for Public Health Education (the accreditation body for schools of public health in US) have revised their competencies framework. The Public Health Agency of Canada has released competencies for the public health workforce [13] and there has been a rapid expansion of schools of public health across the nation. While European schools of public health are developing competencies [14] with a view to educational harmonisation under the Bologna Process, new schools are being developed in India, and there is a national curriculum review process underway in China, where there has been rapid expansion of new schools of public health over the last five years.

At the same time, professional bodies have begun to introduce and or to debate the introduction of various kinds of credentialing arrangements. The US has introduced exams for voluntary credentialing. An international consensus meeting on health promotion competency was held in Galway, Ireland in 2008 [15]. The NZ and UK Faculties of Public Medicine have established mutual recognition arrangements. In Australia, the Faculty of Public Health Medicine introduced an exam based on a competency framework [16], while the Australian Epidemiology Association has debated credentialing, and the Australian Health Promotion Association has been developing its own competency framework [17]. In March 2009, the Department of Human Services (Victoria) published a report on competencies and the health sector and setting standards across the health workforce and education principles [18]

## Workforce planning and capacity development – whose role?

The current situation in Australian is that health workforce policy – including the field of public health is – effected through a combination of government purchasing from the health sector, and market-based education from the education sector. In theory, public health programs exhibit all the characteristics of a public good, and are dependent largely on government investment. As such, it is reasonable that government should be purchasing public health education, in order to produce the workforce required. There is, however, little coordination between the Commonwealth and the states/territories in coordinating their investments, or in projecting workforce requirements.

Beyond PHERP, the public health capabilities and capacity of the health workforce have also been improved through other kinds of government investment (such as through the recently established schools of rural health) and through the efforts of the professional and academic institutions (i.e. incorporating public health content and skills into the curriculum of the clinical workforce). However, the effectiveness of including some public health curriculum into undergraduate medical, nursing, and health sciences education is largely unknown.

Unfortunately, there is little systematic information available about the outcomes and career trajectories of most MPH students and other graduates. For example, it is not known the extent to which such students are seeking career change, or just upskilling. It is also not known the extent to which graduates have proactively shifted into working more full time in the field of public health, or are merely using their public health knowledge and skills in existing positions. Anecdotal information suggests that 'all of the above' are occurring, with the expansion of employment opportunities in a wide range of relevant settings, including Divisions of General Practice, local government, non-government organisations. In 2004 a survey was undertaken of 655 current and alumni MPH students in Victoria. Most students agreed that the MPH training was relevant to their jobs and prepared them for public health positions. However, only 71 per cent of students thought that the MPH prepared them for practice, while half of this number said that it prepared them for research [19]. A series of ANAPHI case studies [20] suggested that PHERP-funded institutions have contributed significantly to developing a workforce capable of addressing such public health challenges as emerging diseases such as SARs, chronic disease, indigenous health and socioeconomic health inequalities [21].

In the absence of a strong government role and leadership in planning and purchasing in a 'public good' field like public health, it becomes the role of the profession to create a market demand, or to reflect on how best to meet the public interest and needs. The articles in this series demonstrate the efforts of the profession and the educators to improve workforce capacity to work in disadvantaged communities (Harris et al) and in low and middle income countries (Patel & Phillips), to institute innovation in education (Bullen & Neuwelt, for New Zealand) and shape the pathways through education (Bennett et al), and to undertake their own planning (Rumbold and Bennett, Fleming et al).

Such a market-based system provides for diversity, as well as problems of sourcing needed expertise (Madden et al). Yet, traditional workforce planning methods of deterministic projections have not been effective in addressing workforce needs (Bolton & Segal). There have been attempts to develop methods for estimating workforce needs [22] as well as workforce planning models specifically for public health [7] – though neither has been sufficiently robust for broader adaptation and uptake.

Within the current policy context, there are some workforce needs which can be anticipated (Lilley & Stewart), while the extent of new public health challenges such as climate change may be less predictable (Ellis et al). In the face of very uncertain futures, currently being impacted on by the greatest global economic downturn to have occurred in the last 50 years, the question for government is how to ensure a sufficiently flexible and adaptive workforce, and how to provide sufficient incentives to academic institutions and the relevant professions to assure a high standard and quality of education in the public interest and for a more capable public health workforce for the future.

## Competing interests

The authors declare that they have no competing interests.
